# A survey of computable biomedical knowledge repositories

**DOI:** 10.1002/lrh2.10314

**Published:** 2022-06-03

**Authors:** Jodyn E. Platt, Anthony E. Solomonides, Philip D. Walker, Philip S. Amara, Joshua E. Richardson, Blackford Middleton, The Mobilizing Computable Biomedical Knowledge Trust and Policy Work Group

**Affiliations:** ^1^ University of Michigan Medical School Department of Learning Health Sciences Ann Arbor Michigan USA; ^2^ Research Institute, NorthShore University HealthSystem Evanston Illinois USA; ^3^ Annette and Irwin Eskind Family Biomedical Library and Learning Center Vanderbilt University Nashville Tennessee USA; ^4^ Center for Health Informatics and Evidence Synthesis RTI International Chicago Illinois USA; ^5^ Mobilizing Computable Biomedical Kinowledge Steering Committee Austin Texas USA

**Keywords:** clinical decision support, computable biomedical knowledge, knowledge management, learning health care, learning health systems

## Abstract

**Introduction:**

While data repositories are well‐established in clinical and research enterprises, knowledge repositories with shareable computable biomedical knowledge (CBK) are relatively new entities to the digital health ecosystem. Trustworthy knowledge repositories are necessary for learning health systems, but the policies, standards, and practices to promote trustworthy CBK artifacts and methods to share, and safely and effectively use them are not well studied

**Methods:**

We conducted an online survey of 24 organizations in the United States known to be involved in the development or deployment of CBK. The aim of the survey was to assess the current policies and practices governing these repositories and to identify best practices. Descriptive statistics methods were applied to data from 13 responding organizations, to identify common practices and policies instantiating the TRUST principles of Transparency, Responsibility, User Focus, Sustainability, and Technology

**Results:**

All 13 respondents indicated to different degrees adherence to policies that convey TRUST. *Transparency* is conveyed by having policies pertaining to provenance, credentialed contributors, and provision of metadata. Repositories provide knowledge in machine‐readable formats, include implementation guidelines, and adhere to standards to convey *Responsibility*. Repositories report having *Technology* functions that enable end‐users to verify, search, and filter for knowledge products. Less common TRUST practices are *User Focused* procedures that enable consumers to know about user licensing requirements or query the use of knowledge artifacts. Related to *Sustainability*, less than a majority post describe their sustainability plans. Few organizations publicly describe whether patients play any role in their decision‐making.

**Conclusion:**

It is essential that knowledge repositories identify and apply a baseline set of criteria to lay a robust foundation for their trustworthiness leading to optimum uptake, and safe, reliable, and effective use to promote sharing of CBK. Identifying current practices suggests a set of desiderata for the CBK ecosystem in its continued evolution

## 1 INTRODUCTION

The production of scientific knowledge is a collective endeavor, contingent on intradisciplinary and interdisciplinary relationships.^1^ (Yet, primary research and subsequent discoveries have long been stored in disparate personal files, department or institutional archives, and journal publications. The world wide web has enabled scientists to upload and share data, information, and knowledge with many more stakeholders.^2^, ^3^, ^4^ Large‐scale research, such as the Human Genome Project, coupled with federal funding mandates have resulted in the creation of numerous online repositories or commons where data, information, or knowledge can be accessed.^5^ This mixing and sharing of data, information, and knowledge creates a shift in the scientific paradigm because it challenges traditional methods of preserving research integrity through peer review, intellectual property, and professional reputation.^6^ Despite a growing awareness and value on transparency in health research, including from federal agencies, the focus to date has been on how research is implemented on the data sources, with less engagement and energy related to sharing computable biomedical knowledge (CBK) resulting in stashes of artifacts dispersed across many individual developers and organizations, and not readily sharable. For example, high quality clinical decision support (CDS) that is available across health systems can optimize care for a broad range of patient populations. To move toward digital healthcare, learning health systems, and the sharing of CBK, we must assess and define methods to promote trustworthy CBK artifacts and methods to share, and safely and effectively use them to improve patient care and health outcomes.

Trust is a necessary condition for the collective endeavor of knowledge building and sharing, just as it is for data aggregation and stewardship.^7^ In both cases, repositories—organizations or other governing entities—manage content, infrastructure, governance, access, and attribution, often on behalf of various contributors and in the public interest.^8^, ^9^, ^10^, ^11^, ^12^, ^13^ For example, trusted health information (or data) brokers are called such because they ensure the integrity of data and appropriate access to information.^14^ Collaborative knowledge engineering and knowledge management, while distinct disciplinary fields, are critical to the knowledge ecosystem and both require formal and informal agreements addressing compliance of knowledge artifacts with accepted industry standards and expected functionality.^15^ While data repositories are well‐established in clinical and research enterprises, knowledge repositories with shareable computable artifacts are relatively new entities to the digital health ecosystem.

Initiatives such as *Mobilizing Computable Biomedical Knowledge* (MCBK) aim to provide guidance to these emergent efforts. MCBK is a volunteer organization whose goal is to promote knowledge building and sharing to achieve a Learning Health System.^16^ The MCBK community has been organized in three themes with Working Groups corresponding to each: Standards & Technical Infrastructure, Trust & Policy, and Sustainability & Inclusion. The fundamental premise on which MCBK's goal is predicated is that knowledge, to be available widely and applied equitably, cannot remain static. To “mobilize” conveys a useful duality of meaning: knowledge must be mobile (portable), must be capable of being moved to be used at the right place and time to inform healthcare decision‐making. The MCBK Trust and Policy Working Group (T&P) aims to identify and analyze the attributes, processes, and procedures that characterize best practices for a biomedical knowledge repository, and how to derive a computable knowledge artifact from paper‐based guidelines and other sources. Our aim is to understand CBK that lies in domains that are commonly accessible and serve a public good.^17^, ^18^

In this article, we report on results of a survey assessing current practices among CBK repositories in the United States. Our focus is on repositories that make up the “knowledge commons” for CBK by generating and/or storing CBK in ways that prioritize sharing rather than being purely proprietary enterprises. In a knowledge commons, communities create rules and institutions that allow for the cooperative governance and equitable management of sustainable shared resources, in this case, knowledge.^18^ Governance policies address the fair and equitable sharing of resources, whether through moral principles or market mechanisms, and lay a foundational layer of trust amongst users and generators of knowledge to sustain a shared ecosystem.^19^

### 1.1 Artifacts in knowledge commons: The four‐layer framework

Biomedical knowledge itself displays functional attributes that impact its utility in informatics infrastructure and “mobility” from one context to another. Boxwala et al developed a framework to describe four “layers” of biomedical knowledge abstraction, typically treatment or diagnostic guidelines in narrative form (Layer 1, L1), abstraction of critical logic and workflow(s) (Layer 2, L2), encoding of key variables and recommendations (Layer 3, L3), and finally in executable software (Layer 4, L4).^20^ This framework helps to measure the development of shareable CBK such as predictive analytic models, quality measures, care management guidelines, and other CDS tools. The L1 to L4 framework is a useful way of characterizing what “product” or artifacts are made available or shared in a knowledge repository. We note that while paper based (L1) artifacts may be generally available often via simple search, they are of course far from computable. We consider the “share‐ability” of CBK in each of these levels (L1‐L4).

### 1.2 A complex CBK ecosystem requires trust and principles of trust to guide it

The abstraction of biomedical knowledge into computable artifacts requires a larger set of actors than traditional paradigms of research and clinical care that involved single researchers and research subjects, or discrete physician‐patient relationships. Today's CBK ecosystem entails management and cooperation between developers, IT implementation teams, EHR and other vendor organizations, among others. This domain is lightly regulated by the FDA and relies on some industry standards (HL7 Arden Syntax, FHIR, CPG on FHIR; OMG BPM+, etc.), and relies on trust between actors to ensure artifacts deliver as expected.^21^, ^22^, ^23^

A number of principles for trusted CBK ecosystems and trustworthy governance have emerged over the past several years.^15^ In the domain of computable knowledge artifacts, the Trust Framework from the Trust Framework Working Group^15^ provides a detailed set of considerations for the computable CDS. They outline nine attributes of trust that should be considered when evaluating the trustworthiness of CDS, including, for example, the evidence base for the product, patient centeredness, competency of the author, provenance, and clarity of the underlying logic. From Information and Library Science, the TRAC Criteria and Checklist^24^ provide an example of not merely approval, but certification of trustworthy repositories of any type of digital knowledge. TRAC details (a) the necessary organizational infrastructure, (b) the process of digital object management, and (c) the required technologies, technical infrastructure, and security measures necessary to meet the standard.

For data, the FAIR Principles for data management and stewardship are highly cited and used in data science and informatics, and have been proposed as key principles for trustworthy CBK.^25^ Similarly, Lin et al's TRUST principles articulate key domains for trusted data repositories: Transparency, Responsibility, User Focus, Sustainability, and Technology.^7^ We use these TRUST principles as the framework for our research as they apply to knowledge repositories as corollaries to data repositories. We do so given that governance frameworks (eg, those of IEEE and the Organisation for Economic Cooperation and Development [OECD]) include transparency and accountability or responsibility among their foundational principles.^26^, ^27^, ^28^ More recent versions include sustainability. If good governance is a foundation for trust, it is appropriate that good governance “not only be done, but to be seen to be done.”^26^ Transparency certainly addresses this point. If transparency is real, then accountability and responsibility can and should be assessed. A commitment to “User Focus” is also widely adopted, although (convenient acronyms notwithstanding) it is better expressed as stakeholder engagement, if only because this then requires an analysis of who the stakeholders are. Under “technology” we understand infrastructure, the structures and processes, not only the tools, that make the service function. As with Donabedian's “structure‐process‐outcomes” scheme, when appropriate structures and processes are in place, the right outcomes should follow—or if they do not, it should be possible to see what has functioned suboptimally (Table 1).^29^

**TABLE 1:** Lin et al's TRUST principles for data organizations^7^

Principle	Definition
Transparency	To be transparent about specific repository services and data holdings that are verifiable by publicly accessible evidence.
Responsibility	To be responsible for ensuring the authenticity and integrity of data holdings and for the reliability and persistence of its service.
User Focus	To ensure that the data management norms and expectations of target user communities are met.
Sustainability	To sustain services and preserve data holdings for the long‐term.
Technology	To provide infrastructure and capabilities to support secure, persistent, and reliable services.

## 2 QUESTION(S) OF INTEREST OR RESEARCH INTERESTS

For this investigation we asked the question, what policies and procedures do CBK repositories in the United States have to convey trust, according to the TRUST framework^**7**^?

## 3 METHODS

### 3.1 Survey development and validation

The survey instrument was developed by the authors and the members of the MCBK T&P. The T&P reviewed multiple existing frameworks, cited above, for trust in CBK and related technologies. We chose to use Lin et al's TRUST principles as the guiding framework for the survey because we were interested in governance of organizational structures (ie, repositories) that manage CBK, comparable to the digital data repositories that were the focus of the TRUST framework.^7^ Additionally, the TRUST principles were developed in the context of biomedical science.

We developed questions to capture information about current practices among CBK repositories related to the TRUST principles: (1) Transparency; (2) Responsibility; (3) User Focus; (4) Sustainability; and (5) Technology. We also developed questions that would provide general descriptive information about the organizations themselves, such as when they were founded and their funding models, and about CBK artifacts, such as where they fit in the L1 to L4 framework for describing layers of biomedical knowledge abstraction. The questions went through multiple rounds of internal editing and were then reviewed and commented upon by members of the MCBK community during both an online question and answer session, and an in‐person committee meeting. The process generated a total of 91 questions that were composed of 60 structured and 31 open‐ended question types, which required 35 minutes on average to complete. The survey is provided as a Supporting Information S1.

### 3.2 Survey distribution

We developed a convenience sampling frame of 48 different organizations that were known to be involved in the development and/or deployment of CBK. We included organizations that were either independent or affiliated with academic institutions, and that had a “public facing” element to their mission. This meant we did not include CBK repositories that were purely proprietary or unique to a single organization or health system. We did not include CBK repositories run by EHR vendors (eg, Epic, Cerner, etc.). Candidate CBK repositories were identified by the MCBK T&P, and MCBK Steering Committee with several iterations of review for completeness. We emailed the survey to 48 knowledge repository representatives, of which 24 either opened the survey link or completed the survey to some extent.

### 3.3 Data analysis

Due to a bimodal distribution of completed responses among the 24 representatives, we set a 40% minimum completion rate as a cutoff for inclusion and arrived at 13 responses in our sample (13 out of 24 responses = 54%). By setting a 40% cutoff we were able to limit data missingness and maximize data quality. We generated summary frequencies for questions capturing information about each organization, and the policies and practices within the five TRUST principles. We then developed a summary table of business practices and organized the results by “yes” votes. The practices were then broken into three categories: “common practices” (at least 7 out of 13), somewhat common (4‐6), or uncommon (3 or fewer). Most respondents requested that their organization names not be published and so we therefore do not list them in this article.

## 4 RESULTS

Twenty‐four respondents accessed the survey, of whom 13 (54%) completed at least 40% of the structured and open‐ended questions. The median time it took to complete was 74 minutes (1.2 hours). The respondents represented organizations that served a broad range of end users. The majority of organizations (n = 9) catered to the healthcare sector and are also in the quality improvement sector. Other organizations indicated that their market sectors included pharmaceutical (n = 3), EHR (n = 3), basic science (n = 3), and research (n = 3). A majority received some form of government funding (n = 9), five indicated they were not‐for‐profit, four indicated receipt of academic funding, and two received for‐profit or commercial funding. Eight organizations made their content free to the public with or without user registration. Nine organizations that make at least some of their knowledge public, either freely available or via a free membership, offer artifacts at L3 (n = 4) or L4 (n = 5) (see Table 2).

**TABLE 2:** CBK repositories by layer (L1‐L4) and user availability

	User availability				
Layers L1 to L4	Free to the general public with registered account	Free to the general public, no registration required	Other (please specify)	Paid members only	grand total
L1—Narrative, human readable only	—	2	—	1	3
L2—Semi‐structured, human readable but with basic logic flow only	—	—	1	—	1
L3—Structured, logic is formally represented (terminology bindings, value sets, expressions) but not computable, for example, clinical quality language only	2	2	—	—	4
L4—Executable, logic is formally represented and computable only	1	1	3		5
Grand total	3	5	4	1	13

We next report key findings from each of the TRUST domains.

### 4.1 Transparency

The Transparency principle is reflected in practices that make services and digital content (ie, CBK) knowable and verifiable.^7^ Eleven of the 13 repositories reported having explicit policies for conveying provenance through practices such as the use of citations or links to original sources, metadata, and use of standard terminologies. Policies for conveying provenance included guidance (tools and tips) for contributing authors, use of wiki environments, and policies posted on repository websites (see Table 3). Eight of the thirteen organizations indicated some form of credentialing requirement for authors. Criteria for authorship included being associated with a known clinical or governmental organization, being a licensed clinician, or being screened or informally vetted by the organization. In at least two instances, inclusion criteria for contributors is posted on repository websites.

**TABLE 3:** Knowledge repository policies for transparency (n = 13)

Policy	Yes	No	No response
Policies for conveying provenance	11	2	0
Policies for credentialed contributors	8	5	0
Implementing, updating, revising, or de‐implementing knowledge products	7	N/A	N/A
Conflict of interest	5	N/A	N/A
Licensing agreements or secondary use rights	6	N/A	N/A

Abbreviation: N/A, not applicable.

### 4.2 Responsibility

Repositories with policies and practices that ensure responsibility for knowledge artifacts have mechanisms for ensuring currency, reliability, and authenticity of CBK artifacts via, for example, adherence to standards, best practices for knowledge management, external review, and certification. Survey questions in this domain focused on best practices for knowledge engineering and management, processes to ensure knowledge artifacts were up to date and validated, and the types of standards used for knowledge representation and citations. Table 4 below details the responses across these items in the knowledge repository survey. Of note, most respondents indicated they were using controlled medical terminology and value sets, and a majority presented their knowledge artifacts in a machine‐readable format. Varying standards were used for other purposes, for example, clinical data models, knowledge representation, logic systems, and citation formats.

**TABLE 4:** Knowledge repository policies for responsibility (n = 13)

Policy	Yes	No	No response
Machine‐readable formats	8	2	3
Knowledge artifacts certified as safe and effective	3	3	7
Include implementation guidance to define resources required to implement in practice	6	5	2
Use of current standards for knowledge representation: Controlled medical terminologies	11	N/A	N/A
Use of current standards for knowledge representation: Value sets	9	N/A	N/A
Use of current standards for knowledge representation: Clinical data models	3	N/A	N/A
Use of current standards for knowledge representation: Knowledge representation formalisms (JSON, CQL, Arden, etc.)	4	N/A	N/A
Use of current standards for knowledge representation: Logic systems (Description Logic, Deontic logic, Datalog, etc.)	3	N/A	N/A
Use of current standards for knowledge representation: Citation standard formats	4	N/A	N/A

Abbreviation: N/A, not applicable.

We also asked what standard citation format was being used and found that there was little commonality in approach in this area as depicted in Table 5.

**TABLE 5:** Citation standards used

Citation format	Count
AMA, APA	1
DOI addresses	1
Export as RIS, XML, CSV, Text using NLM style	1
FHIR citation resource	1
NA	2
No response (NR)	7

Lastly, with respect to Responsibility and which certifying bodies were used to certify knowledge artifacts and ensure the authenticity and integrity of data holdings and for the reliability and persistence of its service respondents some respondents answered an open‐ended question. One stating the VSAC is designed based on HL7 standards, and another stating that the NIH Interagency Modeling and Analysis Group's “10 Simple Rules with Conformance Rubric” was used to check knowledge artifacts,^30^ otherwise no certifying body was mentioned by respondents.

### 4.3 User Focus

CBK Repositories following the principle of *User Focus* have practices that ensure they are meeting the needs and expectations of their user communities, including, for example, patients and clinicians.^7^ Most respondents indicated that when using the CBK an end‐user would have an opportunity to provide feedback to the repository itself, which then could update the artifact as appropriate, or inform the CBK authors. Similarly, end users could ask about appropriate use of the CBK, as well as in some cases ask other users of the same CBK in different settings. A minority of CBK repositories indicated a license was required to use the CBK artifacts. (see Table 6 below and discussion of Business Practices associated with CBK Knowledge Repositories).

**TABLE 6:** Knowledge repository policies for User Focus (n = 13)

Policy	Yes (out of 13)	No	No response
Updates per user feedback	9	2	2
Allows user questions about use	5	7	1
Enables users to ask each other questions	6	6	1
Requires end user license prior to use	6	4	1
End user agreement indemnifies knowledge creators from users	4	1	8

### 4.4 Sustainability

Sustainability is an important domain of trust because it conveys a sense of long‐term preservation and access to the repository's content and platform.^7^ The Sustainability questions inquired about governance structures and sustainability planning. In their free‐text responses, five respondents post their governance structures on their websites. Further review of the webpages described their governing bodies as an editorial board or steering committee primarily made up of volunteers from academia, government, or industry. Based on this review, we found that some knowledge repositories may have additional sub‐committees or advisory boards to report to the main governing body depending upon the size, scope, and breadth of the organization. Of note is that one knowledge repository stated it utilized a user honor system rather than having a governing body. Only two of the 13 respondents stated their governing bodies involved patients or patient advocates. As for sustainability, six organizations had plans ranging from 5 to 10 years. One commercial entity indicated it had developed a decades‐long plan for financial sustainability. Of the remaining seven, two do not have sustainability plans and five did not answer. Interestingly, only four indicated planning five or more years in advance, the remaining did not respond. Six respondents had policies or procedures to ensure accuracy of the content, which addresses the issue of preservation and long‐term sustainability. Eight respondents required attribution of the knowledge artifacts (Table 7).

**TABLE 7:** Knowledge repository policies for sustainability (n = 13)

Policy	Yes	No	No response
Posts a governance structure description	5	3	5
Includes patient voices	2	6	5
Policies and procedures for accuracy	6	4	3
Policies and procedures for attribution	8	2	3
Has a sustainability plan	6	2	5

### 4.5 Technology

A high degree of technological functionality helps to instantiate trust with end users by providing appropriate infrastructure to ensure the capabilities associated with the principles of Transparency, Responsibility, User Focus, and Sustainability.^7^ Eleven respondents indicated having search technology that utilizes keywords or controlled vocabularies, nine of which enable users to search for past or related versions. Others are accessible through an administratively assigned user role. Hyperlinks were provided to past or related versions of artifacts (n = 2), supporting references (n = 10), and/or help materials (n = 8). Nine repositories have systems which enable tracking of product changes and updates over time. The availability of APIs to the CBK repositories varies. Of the nine, five offer theirs for free, three are fee‐based, and one has free and fee‐based APIs. Nine repositories have systems, which enable tracking of changes and updates (Table 8).

**TABLE 8:** Knowledge repository policies for technology (n = 13)

Policy	Yes	No	No response
Search technology with key or controlled terms	9	2	1
Searchable past or related versions	9	1	3
Links to supporting references	10	1	2
Search help materials	8	2	3

## 5 DISCUSSION

We surveyed 13 knowledge repositories to identify the policies and procedures they have in place that can convey trust in their knowledge products based on Lin et al's (T)ransparency, (R)esponsibility, (U)ser Focus, (S)ustainability, and (T)echnology Principles for data organizations.^7^ This is, to our knowledge, the first survey to attempt such an undertaking. Table 9 displays a summary view of the results, highlighting common (occurring in 7 or more repositories), moderately common (occurring in 3‐6 repositories), and uncommon (occurring in 2 or fewer repositories) practices to better discuss their implications for the current landscape of knowledge repositories as well as highlight areas for improvement.

**TABLE 9:** Practices and policies associated with computable biomedical knowledge repositories

TRUST principal	Common practices (n) (≥7)	Moderately common practices (n) (3‐6)	Uncommon practices (n) (≤2)
Transparency	Policies for conveying provenance (n = 11) Policies for credentialed contributors (n = 8) Metadata is associated with—date the knowledge product was originally published (n = 12) Metadata is associated with—last reviewed (n = 9) Metadata is associated with—references to the evidence base(s) (n = 11) Metadata is associated with—citation(s) (n = 11) Posted procedures describe—implementing, updating, revising, or de‐implementing knowledge products (n = 7)	Metadata is associated with—known limitations, restrictions, or exclusions to any given evidence (n = 6) Posted Procedures describe—posted Procedures describe conflict of interest (n = 5) Posted procedures describe licensing agreements or secondary use rights (n = 6)	Metadata is associated with—user history (n = 2) Metadata is associated with—feedback (n = 2)
Responsibility	Computable knowledge stored is in machine readable formats (n = 8) Current standards use—controlled medical terminologies (n = 11) Current standards use—value sets (n = 9)	Knowledge repository include implementation guidance (n = 6) Current standards use—knowledge representation formalisms (n = 4) Current standards use—citation standard formats (n = 4) Current standards use—clinical data models (n = 3) Current standards use—logic systems (n = 3) Knowledge products are developed in compliance with best practices (n = 3)	N/A
User Focus	Knowledge products updated based on user‐provided feedback (n = 9)	Allow users to ask questions or provide feedback to one another/user forums (n = 6) Allow for users to ask questions about an artifact's context of use (n = 5) Require end user licensing agreement to use artifacts (n = 6) EULA indemnify the author/publisher/vendor (n = 4)	N/A
Sustainability	Require user attribution of artifacts used in future products (n = 8)	Post a description of its governance structure (n = 5) Quality control policies or procedures in place (ensure the correctness or accuracy of artifacts) (n = 6) Sustainability plan in place (n = 6)	Patients included in governance decision making (n = 2)
Technology	Make the knowledge products accessible with search technology (n = 9) Help materials made available to inform users how knowledge is findable (n = 8) There is system to tracks updates and changes to the products over time (n = 9) Knowledge repository offers 1 or more APIs (n = 9)	Past versions or artifacts searchable (n = 6) Artifacts provide linkable access to supporting references (n = 6)	N/A

Abbreviations: EULA, end user license agreement; N/A, not applicable.

Taken in summary, many of the practices and policies we surveyed amount to “common” business practices when organized within the TRUST framework, and all the organizations to different degrees adhere to policies that convey forms of TRUST. Today's knowledge repositories commonly convey *Transparency* by having policies pertaining to provenance, credentialed contributors, and explicit metadata such as when knowledge was last reviewed or explicit citations to references. They also convey *Responsibility* upon their knowledge products by adhering to standards per value sets and controlled vocabularies such as LOINC or SNOMED CT. Interestingly, a majority of the repositories make their knowledge in formats that are machine executable (L4) and are freely available to the public with or without online registration. This demonstrates a high level of sophistication in knowledge formats and enhances responsibility because of compliance with national standards either as regulatory mandates, or best practices. Repositories also report having *Technology* functions that enable end users to verify, search, and filter for knowledge products, which conveys trust to users. Yet these “common” business practices do not comprise a uniform approach to conveying trust in CBK, which would be optimal.

Less common than policies and practices for *Transparency, Responsibility, and Technology* across the repositories are *User Focused* procedures that enable consumers to know whether end user licensing is required (n = 6) or ask questions about the use of knowledge artifacts (n = 5) or convey information about end user licensing agreements (EULA). Pertaining to *Sustainability*, less than a majority post their governance structures or describe their sustainability plans. Importantly, few organizations (n = 2) publicly describe whether patients play any role in their decision‐making governance, and if so, then how? Similarly, for *Transparency*, only 5 self‐reportedly posted procedures around conflict of interest with knowledge products, and only 2 organizations each of the 13 associate metadata with user history or feedback.

These results therefore suggest that CBK repositories formulate their knowledge artifacts in ways to make the clinical or research knowledge transparent and address information needs that end users might have. However, these organizations may not be actively involving end users or patients themselves in helping to govern the operations or contribute to long‐term sustainability plans, which may strengthen trust in the organizations and their products. As issues pertaining to health equity and social determinants of health are rightfully gaining emphasis from national to local levels, engaging and activating patients in knowledge curation takes on critical importance.

We used the TRUST principles from Lin et al as the central organizing framework for this survey. We believe that the Principles have been an appropriate framework for organizing the survey questions directed at repositories within the CBK ecosystem. The strengths of our approach is that the TRUST framework is modeled on CoreTrustSeal certification,^31^ which in turn is focused on data repositories focused primarily around natural sciences (oceanography, geology, etc.). The CBK field lacks such centralized certification of knowledge artifacts, and the findings from this article demonstrate that future considerations for such a certifying body may be warranted or even welcomed.

To our knowledge, this has been the first survey to gauge the policies and procedures among knowledge repositories in the CBK landscape, setting the stage for several future areas of inquiry. For example, future work should review this survey to evaluate how repository policies may evolve over time. Other studies should continue the descriptive nature of this study, extending analysis consider the relationships between organizational structures for data and knowledge, the dimensions of validity and replicability/repeatability of research and other CBK objects, and the use of CBK in implementation in learning health systems and related enterprises. Public and end‐user perceptions of trust in L4 CBK (eg, artificial intelligence for CDS) support development of “Product Information Labels” to communicate trustworthiness will also be important in establishing appropriate frameworks for trust in CBK. Future research should also advance the findings of this survey to create tools such as a CBK Repository score for compliance with best practices and consider other models for CBK that emerge from entities that enable distributed query mechanisms, such as the Atlas system for the Observational Health Data Sciences and Informatics (OHDSI) research network or The Cancer Genome Atlas (TCGA), and its tools that apply Machine Learning and other techniques to generate and accelerate discovery.

### 5.1 Desiderata for computable biomedical knowledge repositories

Our survey identifies a few practices that are relatively common while others are scarcer. Synthesizing these findings suggests gaps and opportunities for the CBK ecosystem. Below we develop these to propose a set of desirable attributes for CBK repositories (see Table 10). These recommendations are not presented in order or priority, but should be considered, evaluated, and updated in both research and practice.

**TABLE 10:** Desiderata for CBK repositories

Domain	Best practice
Transparency	Policies for conveying provenance Policies for credentialed contributors Knowledge management meta‐data (sources/citations, publication date, updates, revision cycle) Implementation and Use Guidance CBK metadata to describe—known limitations, restrictions, or exclusions to use any CBK Declarations by all authors and sources of potential conflict of interest CBK with stated procedures describing licensing agreements or secondary use rights (if any) CBK with standard preferred citation formats CBK designed and implemented for use with standard clinical data models CBK end‐user comments are accessible, searchable CBK that is certified by an external agency to the CBK repository and deemed safe and effective for use
Responsibility	CBK stored in current standard(s) machine readable format CBK encoded with current terminology standards, value sets, expressions CBK encoded with current standard knowledge representation formalism(s) CBK products are developed in compliance with best practices for knowledge engineering
User‐focus	CBK Repositories promote end user‐feedback, and visibility on other CBK artifact implementations CBK updated based on user‐provided feedback CBK user with clear end user licensing agreement (free, or paid) CBK EULA clearly states the rights and responsibilities of the author or publisher of CBK CBK enhances health outcomes and improves health equity
Sustainability	CBK conveys attribution of artifacts CBK repository clearly states governance structure CBK repositories include patient and public advocates in governance structure CBK development includes appropriate quality control/quality assurance procedures to assure appropriate, safe, and effective use CBK repository has sustainability plan in place (both public and private repositories)
Technology	CBK repository supports version control CBK is FAIR—findable, accessible, interoperable, and reusable CBK meta‐data to track updates and changes over time CBK Repositories offer an API to access or use CBK (run‐time)

### 5.2 Limitations

This study has limitations that are important to note. First, we recruited from a convenience sample of organizations and their representatives, although we vetted the invitation list multiple times with multiple stakeholders. Second, not all respondents filled out all the questions and so to include/exclude respondents and maintain data quality we decided on a cutoff of 40% completion; results and takeaways may have changed if a greater proportion of respondents had completed the survey. Third, we worked to make the survey as efficient as possible but recognize the length of the survey required extended times for some to complete. We anticipate this first survey will help us to refine and field a second, more parsimonious survey in the future. Lastly, we did not go back to respondents to double‐check answers (ie, “member check”) due to some respondents not wanting to be contacted. We none the less provide a benchmark view that can be used to assess future studies that chart developing landscape of CBK repositories that are fostering a new wave of CBK available to a variety of end users.

## 6 CONCLUSION

In the digital transformation of US and global healthcare we have made significant strides in making standardized clinical data interoperable via industry and regulatory initiatives such as FHIR and the 21st Century Cures Act. Building upon this momentum, we suggest similar thinking needs to be applied to CBK to assure it is FAIR, upholds the principles of TRUST, and may be sustainably accessed and used to improve care delivery by end‐user clinicians, and ultimately by patients. Attention to the policies and practices of CBK repositories is critical to a mature ecosystem that moves beyond ad hoc solutions ill‐equipped to keep pace with the rapid growth of CBK artifacts, increasingly engaging Machine Learning and Artificial Intelligence methods, in both public and private spheres. Higher levels of maturity in the CBK ecosystem will power learning health systems and improve CDS systems, catalyze translational research, and facilitate the spread of appropriate care strategies. This is the first article to comprehensively assess the knowledge ecosystem for the policies and practices that lay the groundwork toward standards for computable knowledge sharing that need to be part of a sustainable knowledge ecosystem. Future investigation and development of the CBK Repository Desiderata proposed here mark an important next step toward a robust and expanded set of research engaging consumers, identifying systems of certification and assurance, and considering additional models for mobilizing CBK.

## CONFLICT OF INTEREST

The authors declare no potential conflict of interest.

## Supporting information


**Data S1** Supporting Information
