# Supplementary material for: A survey of computable biomedical knowledge repositories
Source: Learn Health Syst. 2022 Jun 3;7(1):e10314. doi: 10.1002/lrh2.10314 (PMC9835044; doi:10.1002/lrh2.10314)
Supplement: Supplementary file 1 — Data S1 Supporting Information [file LRH2-7-e10314-s001.docx]

MCBK - Trust and Policy (Sept 2020)

Start of Block: Intro

Q1 Thank you for agreeing to take this survey from the Mobilizing Computable Biomedical Knowledge Trust and Policy Working Group (MCBK T&P). The MCBK T&P is a volunteer group that is interested in understanding the policies and practices that govern knowledge repositories and the organizations that may enable the development and dissemination of computable biomedical knowledge, either currently or in the future.    This survey asks questions about your knowledge repository and the services and products you offer. When we ask about “knowledge products” this refers to a wide range of computable biomedical knowledge. Examples include computable guidelines, clinical decision support, risk calculators, terminologies, value sets, knowledge artifacts, and other resources. We will ask you to specify the type of knowledge product(s) you make available in your knowledge repository.

We will also ask you about policies, practices, and the metadata schema for your repository that promote trustworthiness - the notion that the knowledge products are safe and effective to use in practice. The final questions ask about the structure of your organization and a little about you.

We expect this survey will take approximately 20 minutes to complete.

Thank you for completing the survey. We very much appreciate your responses and look forward to learning from you!

End of Block: Intro

Start of Block: About the organization

Q2 What is the name of your organization’s knowledge repository?

________________________________________________________________

Q3 What year was the knowledge repository made available?

▼ 2020 ... 2000 or before

Q4 What is the URL of your knowledge repository?

________________________________________________________________

Q5 What category best describes the content in your knowledge repository? (Select all that apply)

- Clinical
- Quality Improvement
- Genomics
- Pharmaceutical
- Basic science
- Billing / Financial
- Other [Please Describe] ________________________________________________

Q6 How would you describe the knowledge products in your repository? (CDS, computable guidelines, etc.)

- Knowledge products in my repository include: ________________________________________________
- Do not know

Q8 To whom is the knowledge in your repository made available?

- Free to the general public, no registration required
- Free to the general public with registered account
- Paid members only
- Other (Please Specify) ________________________________________________

Q9 Using the Boxwala et al. “Multi-layered Knowledge  Framework” (L1 to L4) for knowledge abstraction and specification, what is the highest layer that you store knowledge in your repository?

- Layer 1 (L1) - Narrative, human readable only
- Layer 2 (L2) - Semi-Structured, human readable but with basic representational logic, e.g. flow chart, decision tree, etc.
- Layer 3 (L3) - Structured, logic is formally represented (terminology bindings, value sets, expressions) but not computable, e.g. Clinical Quality Language
- Layer 4 (L4) - Executable, logic is formally represented and computable (Please specify compute environment) ________________________________________________
- Do not know

Q10 Does your knowledge repository provide links to original evidence sources, including any real world evidence (data commons) from which knowledge may be derived?

- Yes
- No
- Not applicable
- Do not know

Q12 Do you make any authoring tool(s) available to your users through the repository?

________________________________________________________________

Q13 What are the top 3 most accessed knowledge products that are available in your repository?

- No. 1 ________________________________________________
- No.2 ________________________________________________
- No.3 ________________________________________________
- Do not know
- Not applicable

Q14 Who are the intended users for your knowledge repository?

- Please write your response in the box ________________________________________________
- Do not know

Q15 Do you have a published article in the peer-reviewed literature describing your knowledge repository?

- Yes (please provide citation) ________________________________________________
- No
- Do not know

End of Block: About the organization

Start of Block: Trust: Transparency

Q16
Addressing Trust

For the following questions, we’d like to know about the policies and practices for your knowledge repository, specifically those that you believe engender trust in your knowledge products. We have divided the questions into sections according to the TRUST principles for digital repositories identified by Lin et al (2020) - Transparency, Responsibility, User Focus, Sustainability, and Technology -  and informed by Richardson et al.’s Trust Framework for publicly accessible clinical decision support knowledge artifacts.

Please remember that we are interested in knowledge products and knowledge repositories, not data repositories. Data repositories comprise content that is to be transformed into knowledge whereas knowledge repositories contain content that has already been transformed from data into knowledge.

Lin D, Crabtree J, Dillo I, Downs RR, Edmunds R, Giaretta D, et al. The TRUST Principles for digital repositories. Sci Data [Internet]. 2020 Dec [cited 2020 Jun 26];7(1):144

Richardson, JE, Middleton, B, Platt, JE, Blumenfeld, BH. Building and maintaining trust in clinical decision support: Recommendations from the Patient‐Centered CDS Learning Network. Learn Health Sys. 2020; 4:e10208

Q17 The following questions are about transparency particularly as it relates to being transparent about specific repository services and knowledge products that are verifiable by publicly accessible evidence.

Q18
Do you have a policy for conveying the provenance of knowledge products in your repository?

- Yes (Please specify how you convey provenance of knowledge products) ________________________________________________
- No
- Do not know

Q19 Does your knowledge repository have a policy that contributors (e.g. issuers of guidelines, clinical prediction rules, value sets, or other logic expressions) are credentialed; for example, clinical board certification, licensure, or other educational qualification?

- Yes (Please specify or comment) ________________________________________________
- No
- Do not know
- Other (please specify) ________________________________________________

Q20 Please indicate what if any metadata gets associated with the knowledge products in your repository (Select all that apply)

- The date the knowledge product was originally published
- The date the knowledge product was last reviewed.
- Any known limitations, restrictions, or exclusions to any given evidence.
- References to the evidence base(s) on which a knowledge product is based (e.g., narrative guidelines and the data supporting those guidelines.)
- Citation(s)
- Usage history
- Feedback
- Other (specify) ________________________________________________
- Do not know

Q21 Does your knowledge repository post procedures that describe any one of the following (Select all that apply)

- Implementing, updating, revising, or de-implementing knowledge products.
- Conflict of interest
- Licensing agreements or secondary use rights
- Other (please specify) ________________________________________________
- Do not know

Q22 In your knowledge repository? Remember that transparency refers to being transparent about specific repository services and data holdings that are verifiable by publicly accessible evidence.

________________________________________________________________

End of Block: Trust: Transparency

Start of Block: Trust: Responsibility

Q23 The following questions are about **responsibility**. Responsibility refers to ensuring the authenticity and integrity of knowledge holdings and the reliability and persistence of services such as knowledge translation and specification, implementation, and special services.

Q24 For any computable knowledge that may be stored in your repository (e.g. CDS artifacts), do you offer machine-readable formats (e.g., JSON, CQL, Arden, etc.) in addition to human-readable formats?

- Yes, machine readable
- Not machine readable
- Do not know
- Not applicable

Q25 The knowledge products in your repository are developed in compliance with best practices for safe and effective implementation as per some certifying body, e.g. ISO.

- Yes (Please specify certifying body) ________________________________________________
- No
- Not applicable
- Do not know

Q26 Does your knowledge repository include implementation guidance that conveys the necessary resources to implement the knowledge product into practice?

- Yes
- No
- Not applicable
- Do not know

Q27 What, if any, current standards does your knowledge repository use for representing knowledge in its products? (Select all that apply)

- Controlled medical terminologies
- Value sets
- Clinical data models
- Knowledge representation formalisms
- Logic systems (Datalog, Description Logic, Deontic Logic)
- Citation standard formats (Please specify commonly used citation standards) ________________________________________________
- Other(s) (Please specify) ________________________________________________

Q28 Is there anything else you would like to tell us about how you promote **responsibility** in your knowledge repository? Remember that responsibility refers to ensuring the authenticity and integrity of data holdings and for the reliability and persistence of its service.

________________________________________________________________

End of Block: Trust: Responsibility

Start of Block: Trust: User Focus

Q29 The following questions are about the **user focus** of your repository. User focus refers to meeting management norms and expectations of target user communities.

Q30 Are knowledge products updated based on user-provided feedback?

- Yes
- No
- Do not know
- Not applicable

Q31 Does your knowledge repository allow for users to ask questions about an artifact’s context of use (e.g., research v. clinical care)?

- Yes
- No
- Do not know
- Not applicable

Q32 Does your knowledge repository allow users to ask questions or provide feedback to one another (e.g., through a user forum)?

- Yes
- No
- Do not know
- Not applicable

Q33 Do you require a EULA (end user licensing agreement) to use your knowledge products?

- Yes
- No
- Do not know
- Not applicable

Q34 Does the EULA indemnify the author / publisher / vendor of the knowledge product(s)?

- Yes
- No
- Do not know

Q35 Is there anything else you would like to tell us about how you promote **user focus** in your knowledge repository? Remember that user focus refers to that the data management norms and expectations of target user communities are being met.

________________________________________________________________

________________________________________________________________

________________________________________________________________

________________________________________________________________

________________________________________________________________

End of Block: Trust: User Focus

Start of Block: Trust: Sustainability

Q68 The following questions relate to **sustainability** as "the ability of [a] repository to provide services over time, and to respond with new or improved services to meet evolving user community requirements.”

Q69 Does your knowledge repository post a description of its governance structure?

- Yes (Please provide link and/or description) ________________________________________________
- No
- Not applicable
- Do not know

Q70 Does your governance structure include patients, patient advocates, or patient voices in any ways?

- Yes (Please describe) ________________________________________________
- No
- Not applicable
- Do not know

Q71 Do you have established policies or procedures to ensure the correctness or accuracy of the products in your knowledge repository?

- Yes (Please describe) ________________________________________________
- No
- Not applicable
- Do not know

Q72 Does your knowledge repository require users to attribute any knowledge artifacts they use in future products?

- Yes (Please describe) ________________________________________________
- No
- Not applicable
- Do not know

Q73 Do you have a sustainability plan for your organization that addresses the financial means to exist over a defined period of time?

- Yes
- No
- Not applicable
- Do not know

Q75 Please describe your sustainability plan.

________________________________________________________________

________________________________________________________________

________________________________________________________________

________________________________________________________________

________________________________________________________________

Q76 How many years far into the future do you plan for?

________________________________________________________________

Q74 Is there anything else you would like to tell us about how you promote sustainability in your knowledge repository? Remember that sustainability refers to the ability of [a] repository to provide services over time, and to respond with new or improved services to meet evolving user community requirements

________________________________________________________________

________________________________________________________________

________________________________________________________________

________________________________________________________________

________________________________________________________________

End of Block: Trust: Sustainability

Start of Block: Trust: Technology

Q36 The following questions are about **technology**. In this context, technology refers to the ability of a repository to provide services over time and to respond with new or improved services to meet evolving user community requirements.

Q37 Does your knowledge repository make the knowledge products accessible with search technology that leverages key terms or controlled vocabularies?

- Yes
- Sometimes
- No
- Do not know
- Not applicable

Q38 Does your knowledge repository make the knowledge products searchable so that users can access any past versions or related versions? Please provide any optional comments with your answer.

- Yes ________________________________________________
- Sometimes ________________________________________________
- No ________________________________________________
- Do not know
- Not applicable

Q39 Do the knowledge products in your knowledge repository provide linkable access to supporting references?

- Yes
- Sometimes
- No
- Do not know
- Not applicable

Q40 Are help materials made available to inform users how knowledge is findable?

- Yes
- No
- Do not know
- Not applicable

Q41 Does your knowledge repository use a system that tracks updates and changes to the products over time?

- Yes
- No
- Do not know
- Not applicable

Q42 If your knowledge repository contains computable knowledge products (e.g. clinical decision support executables), does it utilize a system to log usage or errors in use, or other tracking measures?

- Yes
- No
- Do not know
- Not applicable

Q66 Does your knowledge repository offer 1 or more APIs?

- Yes
- No
- Do not know

Q67 If you do offer APIs, are they free or paid/fee-based?

- Free
- Paid/ fee-based
- Some paid, some free
- We do not offer APIs
- Do not know

Q43 Is there anything else you would like to tell us about how you promote **technology** in your knowledge repository? Remember that technology refers to the ability of [a] repository to provide services over time, and to respond with new or improved services to meet evolving user community requirements.

________________________________________________________________

________________________________________________________________

________________________________________________________________

________________________________________________________________

________________________________________________________________

End of Block: Trust: Technology

Start of Block: About your organization

Q45 **About your Organization**


 Please answer each question below as best you can. We appreciate any additional comments or clarifications specific to your organization.

Q46 What market segment(s) best describes your organization? This may differ from the knowledge repository of interest. (Select all that apply)

- Healthcare
- Quality Improvement
- Genomics
- Pharmaceutical
- Basic science
- Financial / Billing
- Consumer Tech
- Electronic Health Records
- Continuing Medical Education
- Other (please specify) ________________________________________________

Q47 What categories best describe the funding sources for your organization? (Select all that apply)

- Commercial, for-profit
- Non-profit
- Academic (private or public)
- Government
- Other ________________________________________________

Q48 Which of the following best describes your core business model(s) for your organization?

- Flat fee (Subscription or paid membership)
- Transactional usage fee (charge per use)
- Grants and contracts
- Government subsidy
- No fee (publicly accessible)
- Other ________________________________________________

Q49 Do you have or offer incentives to encourage participation in the knowledge repository?

- Yes (please describe) ________________________________________________
- No
- Do not know
- Not applicable

Q50 Do any of the following privacy policies apply to your organization? (Select all that apply)

- HIPAA Compliance as a Covered Entity
- HIPAA Compliance under a Business Associate Agreement
- Compliance with the European General Data Protection Regulation (GDPR)
- Compliance with the California Consumer Privacy Act (CCPA)
- Other (please specify) ________________________________________________
- Not applicable
- Do not know

Q51 What, if any, copyright standards does your organization use? (Select all that apply)

- Creative Commons Licensing
- Open Access Guidelines
- US Copyright / Patent Law
- EU Copyright/ Patent Law
- Other (please specify) ________________________________________________
- Do not know
- Not applicable

Q52 Can you estimate the yearly gross revenue for the knowledge repository business, or related business unit, at your organization?

- Less than $100,000
- Between $100,000 and $499,999
- Between $500,000 and $999,999
- $1,000,000 - $24,999,999
- Greater than $25,000,000
- Does Not Apply
- Decline to Answer

Q53 What is the estimated annual number of users for the knowledge repository?

- 101 - 1000
- 1001 - 5000
- 5000 - 10,000
- >10,000
- Do Not Know

Q54 What is the estimated number of full-time staff dedicated to the knowledge repository?

- 1 - 5
- 6 - 10
- > 10
- Do Not Know

End of Block: About your organization

Start of Block: About you

Q56 **About you**

Q57 What is your job title?

________________________________________________________________

Q58 What role do you play in service or product development?

- Make ultimate decision
- Participate in ultimate decision
- Contribute alternatives for discussion in a regular forum (e.g. dept. meeting)
- Contribute ideas through a website or drop box
- None
- Prefer not to say

Q59 What is your highest degree obtained?

- MD, JD, DNP
- PhD
- MSN, MSc, MPH, MLS
- BSN
- Other Bachelor's degree or equivalent
- Other (specify) ________________________________________________

Q60 What is your name? We ask you to give your name only to verify that we are receiving responses from a bona fide contributor to this survey. Your name will not be included in any results or other document without your explicit consent or specific permission, depending on the occasion

________________________________________________________________

Q64 Do you have any comments or questions about this questionnaire that you would like to share?

________________________________________________________________

Q65 The answers you provide will remain anonymous and reported in aggregate. That said, would you be willing to be identified?

- My organization can be identified by name
- I can be identified by name
- No

End of Block: About you

Start of Block: Final comments

Q80 Thank you for taking this survey. If you have any questions or comments would like to tell us, please send an email to **MCBK-Policy@umich.edu**

End of Block: Final comments
